# Electroacupuncture upregulates ERK signaling pathways and promotes adult hippocampal neural progenitors proliferation in a rat model of depression

**DOI:** 10.1186/1472-6882-13-288

**Published:** 2013-10-28

**Authors:** Liu Yang, Na Yue, Xiaocang Zhu, Qiuqin Han, Qiong Liu, Jin Yu, Gencheng Wu

**Affiliations:** 1 Department of Integrative Medicine and Neurobiology, Institute of Acupuncture Research (WHO Collaborating Center for Traditional Medicine), State Key Lab of Medical Neurobiology, Institutes of Brain Science, Shanghai Medical College, Fudan University, Shanghai 200032, P.R. China

**Keywords:** Electroacupuncture, Depression, Neural stem cell, p-ERK, Hippocampal dentate gyrus, Microdialysis

## Abstract

**Background:**

In this study, we investigate the proliferation of adult neural stem cells (NSCs) in a chronic unpredictable stress (CUS) rat model of depression, the effects of electroacupunture (EA) on depressive-like symptoms and the corresponding signaling pathways.

**Methods:**

SD rats were subjected to 4 weeks of CUS to induce depressive-like behaviors. EA was performed at the Du-20 (Bai-Hui) and GB-34 (Yang-Ling-Quan) acupoints. Rats were injected with BrdU and the brains were cut into sections. Double-labeling with BrdU/Sox2 and p-ERK/Nestin was performed to demonstrate the in vivo proliferation of adult NSCs in hippocampus and ERK activation in NSCs. Hippocampal microdialysates of different groups were collected to observe the in vitro effects on NSCs.

**Results:**

After 8 treatments, EA generated a clear antidepressant effect on the stressed rats and promoted the NSC proliferation. ERK activation might be involved in the antidepressant-like effects of EA treatment. Hippocampal microdialysates from EA-treated stressed rats influenced NSCs to form larger neural spheres and exhibit higher p-ERK level in vitro, compared to the untreated stressed rats. Meanwhile, the antidepressant-like effects of EA involved contribution from both acupoint specificity and electrical stimulus.

**Conclusions:**

EA might interfere with the hippocampal microenvironment and enhance the activation of ERK signaling pathways. This could mediate, at least in part, the beneficial effects of EA on NSC proliferation and depressive-like behaviors.

## Background

Electroacupuncture (EA) is one of the most frequently used therapeutic modalities for mental disorders, particularly in the East [1,2]. A growing number of studies have demonstrated the antidepressant-like effects of EA [3,4]. However, the neural mechanisms underlying the antidepressant-like effects of EA on depression remain complicated. One potential mechanism which has been repeatedly mentioned is that EA is beneficial to adult neural stem cell (NSC) survival and proliferation, which underlies antidepressant-like effects of EA or classical antidepressants [5,6]. Nevertheless, the signaling pathways involved in the effects of EA on the proliferation of NSCs remain largely unexplored.

Various growth/neurotrophic factors, such as nerve growth factor (NGF), brain-derived neurotrophic factor (BDNF) and glial cell line-derived neurotrophic factor (GDNF), have been shown to mediate the survival of neurons and the proliferation of NSCs [7,8]. While several studies have indicated that EA raises the levels of these growth/neurotrophic factors [9,10], the extracellular signal-regulated kinase (ERK) has been demonstrated to mediate the effects of these factors on adult neurogenesis [1,10]. Therefore, it is possible that EA improves the depressive-like behaviors and activates NSCs through signaling pathways including ERK.

At present, we explored the proliferative effect of EA on adult NSCs, and then investigated the level of phosphorylated extracellular signal-regulated kinase (p-ERK) of hippocampal NSCs in a chronic unpredictable stress (CUS) rat model of depression. In addition, to determine whether EA results in microenvironment change that is conducive to NSC proliferation in the dentate gyrus (DG), we observed the effects of microdialysates from the DG of CUS rats with/without EA treatment on the proliferation of NSCs in vitro. The results exhibited that the growth of neurospheres and p-ERK levels in NSCs were up-regulated by microdialysates from the EA-treated stressed rats. Taken together, the antidepressant-like action of EA on the CUS model might be associated with the extracellular microenvironment of hippocampal NSCs.

## Methods

### Animals

The experimental protocol and procedures used in this study were both in strict accordance with the National Institutes of Health Guide for the Care and Use of Laboratory Animals and approved by the Experimental Animal Ethics Committee of Shanghai Medical College (Fudan University). Sprague–Dawley rats (male, 220 g) were housed in individual cages, at 20–22°C with a 12/12 h light/dark cycle and with free access to food and water.

### CUS-induced depression model

SD rats were subjected to 4 weeks of CUS to induce depressive-like behaviors [4,11]. Seven types of stressors were used (40-h water deprivation, 40-h food deprivation, revered light–dark-cycle, 5-min hot environment, 5-min swimming in cold water, 30-min cage shake and wet cage) in a semi-random order. Rats were exposed to one or two stressors every two days (the next stressor appeared on the next day or two days later). Every type of stressors would appear at least once in the first 2 weeks. Nineteen times of stressors were used in all.

### EA delivery

The stressed rats in EA group started to receive EA on day 15 and were treated for 2 weeks (the latter part of the CUS period, 8 times, once every other day). EA stimulation was performed at 'Bai-Hui’ (Du-20) (+) and 'Yang-Ling-Quan’ (GB-34, the right side) (-) acupoints. These two acupoints have been applied and showed some antidepressant effects in our preliminary and other studies [12-15]. The rat was moderately bound by a piece of self-made clothing and hung approximately 0.15 m high, under halothane anesthesia. A pair of stainless steel needles was connected to the output terminals of the EA apparatus (LH202H, HANS Electronic Apparatus, 2\100Hz, 0.3 mA, lasted for 30 min). Taken together, the rats were divided into three groups (n = 7 ~ 8 for each group): Normal, Model (i.e. CUS) and EA (i.e. CUS + EA) groups.

### Behavioral tests

We used the forced swimming test (FST) and elevated plus-maze (EPM) to determine the depression degree, as extensively observed in many studies. The FST was performed on the first day of every week. Briefly, the rats were individually placed in a glass cylinder (30 cm of diameter) containing 25 cm of fresh water. A fifteen min adaptation of the water was performed one day prior to the first test. The total durations of immobility time and climbing time within the 5 min test period were recorded. An increased immobility time or decreased climbing time was indicative of depressive-like behavior. In the EPM test, the rats were allowed to move freely for 5 min in the apparatus. Percent entries into and time in open arms were related to fear of novelty. Measures were: (1) percentage open-arms entries ([open-arms entries/(open-arms + enclosed-arms entries)] × 100%) and (2) percentage open-arms time ([open-arms time/(total-arms time)] × 100%). We tested these two indices before CUS, in the second, third and fourth week of CUS by different sets of animals.

### BrdU injection

To examine the proliferation of hippocampal progenitor cells, rats were injected with 5-bromo-2-deoxyuridine (BrdU; 100 mg/kg, i.p.) on the final day of the 4-week period and were sacrificed 24 h after injection. The short survival time following BrdU injection allowed us to determine the effect of different manipulations on the proliferation rate of progenitor cells.

### Perfusion and tissue storage

Rats were anesthetized with chloral hydrate (350 mg/kg) and transcardially perfused with 200 ml normal saline followed by 300 ml 4% paraformaldehyde (PFA) in 0.1 M phosphate-buffer (PB, pH 7.4). The brains were removed from the skulls, and postfixed with 4% PFA in 0.1% PB for 24 h at 4°C. Following post-fixation, the brains were immersed in 30% sucrose in PB for 48 h at 4°C, and then, serial coronal sections of the brains were cut through the entire hippocampus into 30 μm sections on a freezing microtome (Leica CM1900, Germany) and stored in cryoprotectant (25% ethylene glycol, 25% glycerol, 0.05 M PB, pH 7.4) at -20°C until use.

### Immunohistochemical analysis

To investigate the proliferation of NSCs in DG, we examined BrdU-labeled Sox2-positive cells in the coronal brain sections. Sox2 is a marker for stem/progenitor cells, and BrdU is used to characterize the cells with mitotic activity. Free-floating brain sections were processed for BrdU immunohistochemistry. The brain sections were incubated for 30 min in 2 M HCl at 37°C, followed by several PBA rinses. After blocked/permeabilized for 1 h in PBS containing 4% normal horse serum, 1% BSA (Sigma-Aldrich, USA) and 0.3% Triton X-100 at 37°C, the sections were incubated with sheep anti-BrdU (1:100, Abcam, UK) and rabbit anti-Sox2 (1:200, Sigma-Aldrich, USA) overnight at 4°C. Next, the sections were transferred into the secondary antibody solution (donkey anti sheep, R-Phycoerythrin conjugated, 1:200, Jackson ImmunoResearch, USA; donkey anti rabbit, Alexa 488 conjugated, 1:200, Invitrogen, USA; Hoechst, 1: 1000, Beyotime, China) and incubated for 1 h in the dark. The sections were then washed 5 times with PBS, covered with a coverslip, and sealed with 75% glycerol.

For Nestin and p-ERK double-staining, the protocol was similar except that HCl is not used and the antibodies was changed to rabbit anti p-ERK1/2 (1:200, Invitrogen, Life Technologies Inc., USA; corresponding secondary antibody: goat-anti-rabbit-Dylight 488, 1:200, Invitrogen, Life Technologies Inc., USA) and mouse-anti-Nestin (1:200, millipore, USA; corresponding secondary antibody: goat-anti-mouse-Alexa 680, 1:200, Invitrogen, Life Technologies Inc., USA).

The hippocampal DG region was examined under a fluorescence microscope or laser confocal microscope. For quantification, we counted the average number of positive cells per section in the DG zone from 3 brain sections of a rat and the data are presented as the average number per rat (n = 5-6 per group). Every two adjacent sections selected were 120 ~ 210 μm apart (distance of 4 ~ 7 sections), and the mean location of the 3 sections was approximately fontanel -3.9 mm.

### Hippocampal microdialysates

After five EA treatments, the hippocampal microdialysates were collected from the rats in Model and EA groups separately. The microdialysis-intracerebral-guide-cannula (Bioanalytical Systems, Inc., West Lafayette, USA) was implanted into the hippocampal DG zone with the following coordinates: 3.9 mm posterior and 1.5 mm lateral to bregma; 3.9 mm ventral to the cortical surface. The guide cannula was fixed to the skull surface with Duralay dental cement. A microdialysis probe with a 2 mm membrane (Bioanalytical Systems, Inc., West Lafayette, USA) was then placed through the guide cannula into the DG region. Physiological saline was applied as the perfusate. The optimal perfusion flow-rate was set at 2.5 μl/min and the sampling time for each rat was 2.5 h (approximately 300–350 μl of microdialysate was obtained, in addition to the loss of the leading portion from the collecting canal).

### NSC culture under the influence of microdialysates

The hippocampus isolated from a postnatal day 0 (P0) SD rat was washed twice and mechanically dissociated into single cells by gentle trituration through a Pasteur pipette in aseptic PBS. The cell suspension was then centrifuged, resuspended, plated in low attachment dishes in NSC-conditioned medium and finally maintained in an incubator (5% CO_2_/air, at 37°C). Components of the media were as follows: DMEM-F-12 medium containing epidermal growth factor (EGF, 20 ng/ml, Invitrogen, Life Technologies Inc., USA), basic fibroblast growth factor (b-FGF, 20 ng/ml, Invitrogen, Life Technologies Inc., USA), and B27 supplements (1:50; Invitrogen, Invitrogen, Life Technologies Inc., USA). The media was changed every 48 h (by centrifugation and resuspension of the spheres). After 9 days of cultivation, we observed the appearance of neurospheres under an optical microscope and detected the expression of Nestin with immunofluorescence (detailed procedures not shown, similarly performed according to universal protocols).

The purified NSCs were then cultured in different media (PBS group: 70% NSC medium + 30% PBS; Model group: 70% NSC medium + 30% microdialysates from CUS model rats; EA group: 70% NSC medium + 30% microdialysates from the EA-treated CUS rats). Before adding into the culture media, the microdialysates of all of the groups were aseptically filtered separately. Then, NSCs were mixed with different media and dissociated by pipette aspiration and inoculated into a 96-well plate (5 ~ 5.5 × 10^3^ cells/120 μl medium/well). After 3 days, another 100 μl of corresponding medium was added to each well and then, after another 2 days, the other 80 μl of corresponding medium was further supplied. The cell viability and growth of the NSC spheres were investigated 48 h after the second supplementation of the culture media.

For cell viability, 100 μl of the cell suspension was transferred into a 96-well plate. CCK-8 (10 μl, Dojindo Molecular Technologies, Inc. Japan) solution was added to each well. The plate was incubated at 37°C for 2 h. The absorbance value at the 450 nm wavelength was measured with an ELx800 Absorbance Microplate Reader (BioTek Instruments, Inc. USA). The experiment was replicated 3 times with quadruplicate samples included in each experiment. The cell viability of the Model and EA groups were converted into a percentage of the PBS group [Cell-Viability_Model_% = (Absorbance_Model_- Absorbance_Blank_)/(Absorbance_PBS_- Absorbance_Blank_) × 100%; Cell-Viability_EA_% = (Absorbance_EA_- Absorbance_Blank_)/(Absorbance_PBS_- Absorbance_Blank_) × 100%].

Another 100 μl NSC suspension was removed to count the number of spheres greater than 100 μm in diameter. This number reflected the NSC-sphere-promoting capacity of the different mediums. In additon, an immunofluorescence assay was employed to detect p-ERK in cultured NSCs in different meida. Briefly, the other 100 μl NSC suspension was transferred to another polylysine-treated 96-well plate and fixed in 4% PFA. The following antibodies: rabbit anti p-ERK1/2 (primary, 1:200, Invitrogen, Life Technologies Inc., USA) goat-anti-rabbit-Dylight 488 (secondary, 1:200, Invitrogen, Life Technologies Inc., USA) were used in immunocytochemical staining. The mean number of immunopositive cells in every well was counted in four non-overlapping visual fields at a magnification of 10× objective lens. The average was calculated for groups of three wells. And the final results are representative of at least three independent experiments.

### Statistical analysis

Results are expressed as means ± S.E.M. One-way analysis of variance was used in most cases to check statistical tendencies. Differences between two groups were analyzed by Student’s *t* test. *P* < 0.05 was considered statistically significant. For NSCs cultures, the numerical data were collected from at least three independent experiments.

## Results and discussion

### Results

#### ***Antidepressant-like behavioral effects of EA on the CUS-induced depression model***

**Figure 1:**
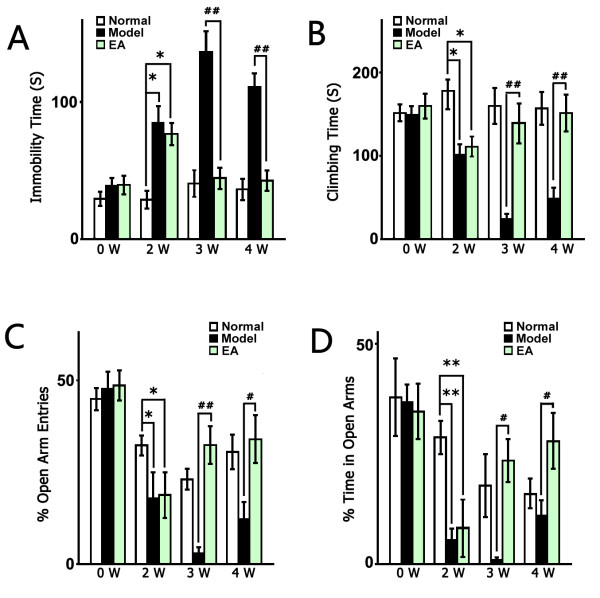
**EA reversed the depressive-like behavior of the stressed rats.** The normal group underwent no stress; the Model group received the chronic unpredictable stress (CUS) for 4 weeks; the EA group received CUS for 4 weeks, as well as EA treatment for the latter part of the CUS (2 weeks). **(A)** The immobility times in the forced swimming test (FST) decreased after 3 and 4 weeks of CUS (1 or 2 weeks of treatment) in the EA group compared with the Model group. **(B)** The climbing time in the FST was reversed to normal levels by EA treatment. **(C)** &**(D)** The percentage of open-arms entries and open-arms time in the elevated plus maze (EPM). The values are presented as the mean ± SE, n = 8 or 18 in each group. Significance of “Model vs Normal” is marked by * (*P* <0.05) or ** (*P* <0.01), and that of “EA vs. Model” is marked by # (*P* <0.05) or ## (*P* <0.01).

Before the CUS procedure, there was no significant difference among the groups according to the FST data. After 2 weeks of CUS, the stressed groups showed significant depressive-like behaviors (increased immobility, F_2, 45_ = 3.874, *P* < 0.05, respectively; and decreased climbing, F_2, 45_ = 4.020, *P* < 0.05, respectively, Figure 1A & B). One to two weeks of EA treatment significantly decreased the immobility and promoted climbing of the stressed rats (immobility time: in the 3^rd^ week F _2, 22_ = 22.90, *P* < 0.01 vs. Model, respectively; in the 4^th^ week F_2, 22_ = 23.10, *P* < 0.01 vs. Model, respectively; climing time: in the 3^rd^ week F _2, 22_ = 13.24, *P* < 0.01 vs. Model, respectively; in the 4^th^ week F_2, 22_ = 10.24, *P* < 0.01 vs. Model, respectively, Figure 1A & B). The EPM test revealed a significant decrease in open arm entries and time spent in open arms after 2 weeks of CUS which indicated the anxiety-like behavior of stressed rats (F_2, 45_ = 2.785, *P* < 0.05 *vs*. Normal, respectively; time: F_2, 45_ = 6.896, *P* < 0.01 *vs.* Normal; respectively, Figure 1C & D). EA treatment also alleviated anxiety-like behavior of stressed rats, as indicated by increased open arm entries and time spent in open arms (in the 3^rd^ week: F_2, 21_ = 8.21, *P* < 0.01 *vs.* Model; F_2, 21_ = 5.511, *P* < 0.05 *vs*. Model, respectively; in the 4^th^ week: F_2, 47_ = 4.569, *P* < 0.05 vs. Model; F_2, 47_ = 2.555, *P* < 0.05 *vs*. Model; respectively, Figure 1C & D).

#### ***EA treatment promotes hippocampal stem cell proliferation in vivo***

**Figure 2:**
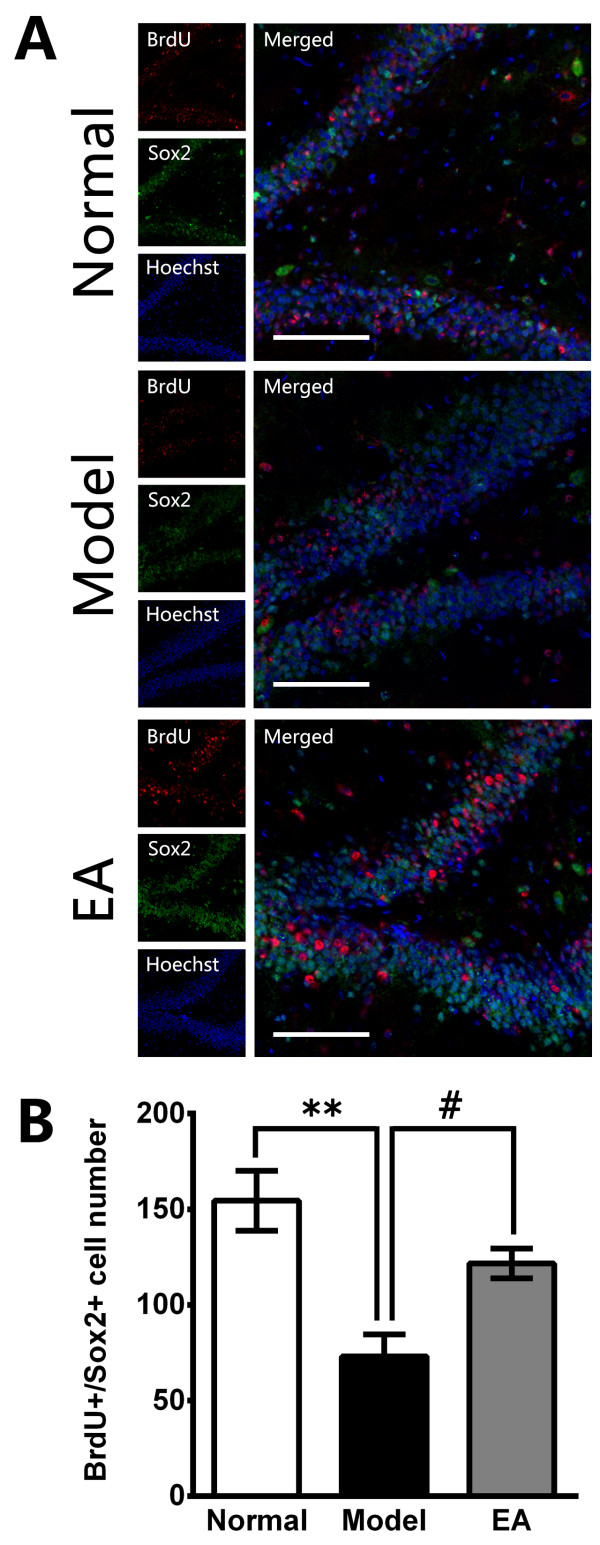
**The effects of EA on stem cell proliferation in the hippocampal dentate gyrus (DG) after 4 weeks. (A)** Representative triple immunofluorescence staining for BrdU + (red), Sox2+ (green) and Hoechst (blue) in the central region of the DG. Scale bar = 100 μm. **(B)** Quantitative representation of Sox2/BrdU double-positive cells in the DG zone (not only those regions in A) per section. EA treatment significantly improved the stem cell proliferation compared with the CUS Model. The values are the mean ± SE, n = 5 in each group. ** *P* <0.01 (Model vs. Normal); # *P* <0.05 (EA vs. Model).

Immunohistochemiscal assay results showed that the number of BrdU+/Sox2+ cells in hippocampus was significantly decreased in the stressed rats compared to that in the normal rats after 4 weeks of CUS procedure (F_2, 15_ = 11.45, *P* < 0.01 *vs*. Normal, Figure 2A & B). Contrarily, the number of BrdU+/Sox2+ cells in hippocampus was significantly increased in EA-treated stressed rats in comparisons to the stressed rats (*P* < 0.05 *vs*. Model, Figure 2A & B).

#### ***EA activates ERK1/2 phosphorylation in hippocampal stem cells in vivo***

**Figure 3:**
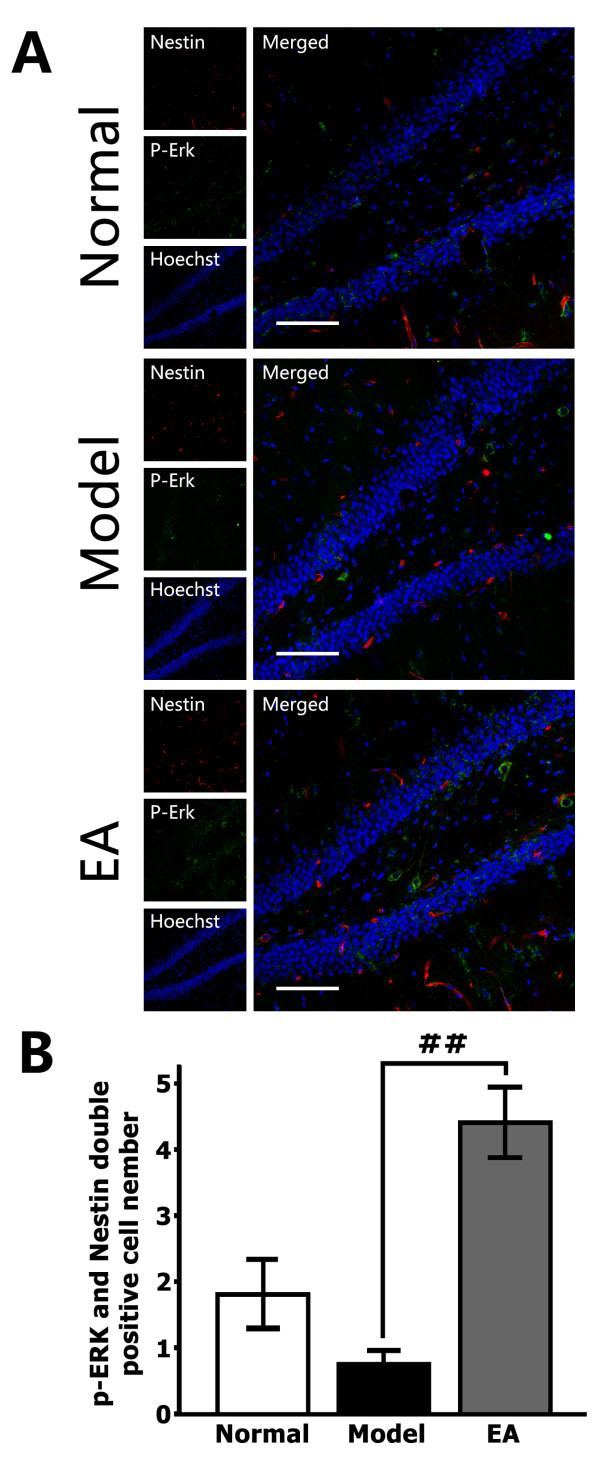
**Induction of ERK phosphorylation (p-ERK) in DG stem cells by EA (8 treatments). (A)** Representative triple immunofluorescence staining for Nestin (red), p-ERK (green) and Hoechst (blue) in the central region of the DG. Scale bar = 100 μm. **(B)** A quantitative representation of Nestin/p-ERK double-positive cell numbers in the DG zone (not only those regions in A) per section. EA treatment significantly elevated p-ERK levels in NSCs in the hippocampal DG of stressed rats. ## *P* <0.01.

Immunohistochemical assay results also indicated a similar relationship between EA and the activation of ERK1/2 in stem cells. The number of p-ERK/Nestin double-positive cells in the DG was greater in the EA-treated stressed rats compared to the stressed rats (F_2, 15_ = 19.10, *P* < 0.01 *vs.* Model, Figure 3A & B). These results show that the MAPK/ERK signaling cascade in NSCs was activated in the EA-treated stressed rats.

#### ***EA microdialysates up-regulated the neurosphere growth and p-ERK level of NSCs in vitro***

**Figure 4:**
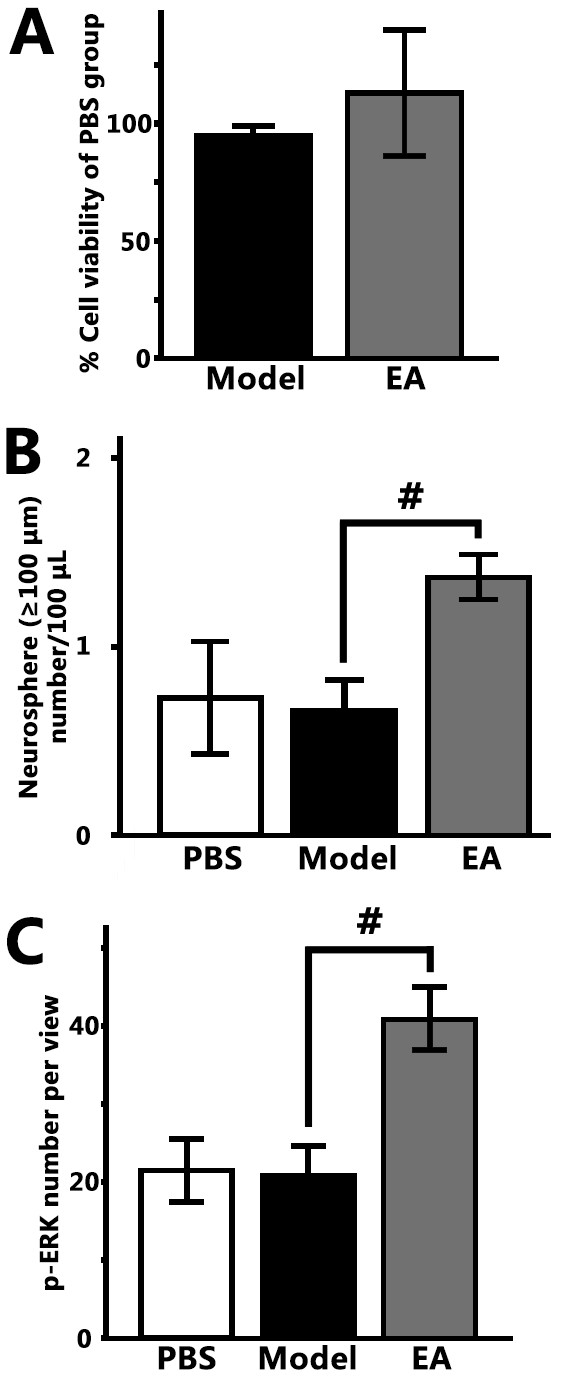
**EA microdialysates enhanced neurosphere growth and p-ERK level of NSCs. (A)** % cell viability of stem cells in the Model and EA medium; **(B)** The formation of big-spheres (diameter ≥ 100 μm) number/100 μl in different mediums; **(C)** The p-ERK + stem cells in an average visual field under the 10× objective lens after 100 μl NSC suspension was fixed to a 96-well plate and stained. The values are presented as the mean ± SE, n = 3, # *P* <0.05 (EA vs. Model).

To determine the microenvironmental effect of EA, the proliferation and p-ERK level of NSCs were checked in different media. And after 7 days of culture in different media, the% cell viability of the EA group was similar to the Model (*P* > 0.05 *vs.* Model, Figure 4A). However, in the EA medium, the number of neurospheres whose diameters were greater than 100 μm was more than that in the Model medium (*P* > 0.05 *vs.* Model, Figure 4B). The number of large neurospheres indicates the tendency to form neurospheres and the maintenance of NSC properties (stem-like properties or stemness). These results suggest there may be dialyzable factors that promote neurosphere formation in the extracellular fluid of the hippocampal DG in the EA stimulated animals. Finally, we analyzed the p-ERK level of NSCs in vitro and found significantly more p-ERK-positive stem cells (visual fields under a 10 × objective lens) in the EA medium (EA vs. Model *P* <0.05, Figure 4C). Therefore, the factors in the hippocampal microenvironment of the EA group may up-regulate the ERK signaling pathway in NSCs. These results are consistent with the in vivo findings and may potentially explain those findings about the antidepressant effects of EA.

#### ***Antidepressant-like effects of EA involved contribution from both acupoint specificity and electrical stimulus***

**Figure 5:**
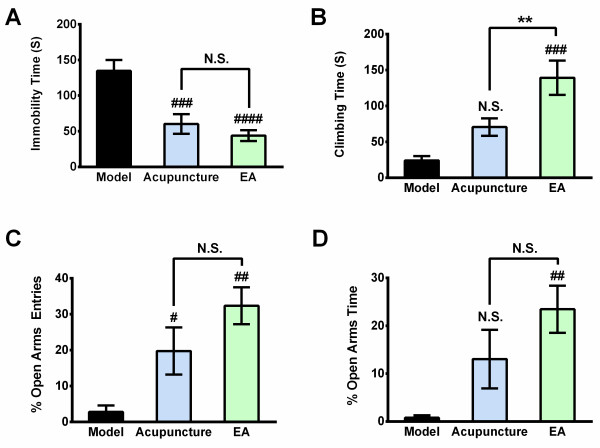
**Antidepressant-like effects of EA involved contribution from both acupoint specificity and electrical stimulus.** Tests were performed after five treatments. **(A)** The immobility times in the FST; **(B)** The climbing time in the FST; **(C)** &**(D)** The percentage of open-arms entries and open-arms time in the EPM. The values are the mean ± SE, n = 8 or 9. # *P* <0.05, ## *P* <0.01 (Acupuncture or EA vs. Model).

To clarify the complicated mechanisms of the Antidepressant-like effects generated by EA, we hypothesized the action of EA involved two parts with separate mechanisms: acupoint specificity and electrical stimulus. Hence, we set a new group named Acupuncture, which was administered similar to the EA group except electrification. Changes produced in this group correspond to the therapeutic effects of physical stimulus at acupoints. After five treatments (began at day 15, after the 3^rd^ week of CUS), Acupuncture significantly exhibited some antidepressant-like effects on the immobility time in the FST and open-arms entries in the EPM, without significant changes in the climbing time and open-arms time. Meanwhile, EA showed much more significant antidepressant-like effects which were evaluated by the significant changes in the immobility time and the climbing time in the FST and open-arms time and entries in the EPM (Figure 5, A: F _2, 22_ = 15.31, *P* < 0.001 Acupuncture vs. Model, *P* < 0.0001 EA vs. Model; B: F _2, 22_ = 12.26, *P* < 0.001 EA vs. Model, *P* < 0.01 EA vs. Acupuncture; C: F _2, 22_ = 9.049, *P* < 0.05 Acupuncture vs. Model, *P* < 0.001 EA vs. Model; D: F _2, 22_ = 6.210, *P* < 0.01 EA vs. Model). These data support that the antidepressant-like effect could be independent of the electrical stimulus and strongly indicate that two factors, acupoint specificity and electrical stimulus, are both making contribution to the behavioral effects of EA.

### Discussion

The major aim of the present study was to investigate the underlying microenvironmental mechanisms involved in the antidepressant-like efficacy of EA in the CUS model of depression. EA exhibited antidepressant-like effects and promoted the proliferation of adult hippocampal NSCs. The underlying mechanism may be related, at least partially, to the up-regulation of extracellular factors in the microenvironment of DG, and then induction of ERK phosphorylation in NSCs.

There have been clinical trials suggesting that EA treatment is an effective therapeutic strategy for depression [4,16]. Consistent with these results, our experiments revealed the antidepressant-like effect of EA in the FST and EPM tests. At the meantime, our findings provided the evidence that an enough therapeutic level of EA included the action from stimulus at specific acupoints and the contribution from the electric current. However, acupoint specificity can also play a lower but significant role, independent of the electrical stimulus. It has been supported that acupuncture of such acupoints as Du-20, GB-34 and othe special acupoints has specific antidepressant-like effect by the traditional Chinese medical theories and many published references [4,12,14,15,17,18]. To date, the underlying mechanism of acupoint specificity is not fully revealed. One possible interpretation could be as follows. Normally, there are more nerves distributed in acupoints than adjacent skin areas. Therefore more nerve receptors are activated during acupuncture treatment at acupoints compared to adjacent skin areas. Electro-acupuncture also produce electrical stimulus to these receptors. The electrical stimulus also contributed to the antidepressant-like effects which were more stabilized and significant. However, exact mechanism merits further study.

Recently, studies on depression have focused on neuroprotection and NSC-proliferation in DG, and an increased number of studies support the hypothesis that acupuncture can be an effective treatment for depression and a rescuer of impaired neurogenesis [19-21]. Our results provide further evidence in supporting this hypothesis.

Although, whether ablation of NSC proliferation or neurogenesis elicits a depression-like phenotype is still highly controversial [21-23], our results are agreement with the relationship between declined NSC proliferation and depression. In addition, there has been broad consensus that the effects of a major antidepressant drugs are closely related to NSC proliferation or neurogenesis [21,24,25]. The present study also confirmed EA, as an alternative therapy for depression, may improve the microenvironment, raise the activation of ERK in NSCs, in turn, up-regulate the declined NSC proliferation and play an antidepressant role. Therefore, this paper may provide some new data regarding the neurobiological bases of the etiology, as well as the recovery, of the depression.

The upregulated level of some growth/neuortropic factors in extracellular fluid might account for the antidepressant effects of EA. And a few of studies have indicated that the levels of these growth/neurotrophic factors were raised in response to EA [1,26-28]. In addition, our work and those of others [29-32], show that relief of the depressive disorder by EA coincides with the activation of the ERK signaling pathway. Intracellular pathways elicited by growth/neurotrophic factors typically converge at the MAP kinase cascade level [33]. In addition to neurotrophic factors, several reports have suggested that EA could regulate the expression level of some anti-apoptotic proteins (e.g., Bcl-2), apoptosis-associated proteins (e.g., Bax, TRPM7), some receptors (e.g., glucocorticoid) and/or some neurotransmitters (e.g., 5-HT) in the hippocampus [29,34,35]. These factors also were involved in the activation of ERK signaling pathway in NSCs [36-38]. EA, in all probability, may activate ERK signaling pathways in NSCs through a variety of routes and improve depressive symptoms.

As the in vivo results showed, EA significantly increased the number of BrdU+/Sox2+ cells and the p-ERK1/2 level accompanying the amelioration of depression. However, the in-vitro-culture experiments revealed that the extracellular fluid of the hippocampus in EA-treated rats did not up-regulate NSC viability. This result may have been caused by the following reasons. First, the most likely reason is that the CCK-8 assay was less sensitive for detecting proliferation, compared with the EdU or BrdU method [39]. Also, the in-vivo role of EA may be underlied by intercellular transmission of information, besides the extracellular ingredients [40].

In addition, our results were consistent with some known references which showed that neurosphere formation (or maintenance of NSC properties) and the ERK signaling pathway in NSCs may be correlative [41,42]. This study suggests that EA might confer a neuroprotective hippocampal microenvironment. Future studies focused on the specific neurochemical changes in the microdialysates might also reveal additional molecular mechanisms.

Finally, the mechanisms mediating the antidepressant-like effects could be the extracellular microenvironment in all probability. Antidepressants (including EA) induced hippocampal cells to establish a proliferation-promoting extracellular microenvironment, and roles in this microenvironment contribute to neurogenesis in the NSCs, highly probably via ERK signaling pathways.

## Conclusions

Taken together, we have shown that the proliferative effects of EA treatment on NSCs may be related to the modulation the ERK signaling pathways in the DG. Microdialysates from the hippocampus of EA-treated rats promoted the in vitro growth of neurospheres and induced ERK-signaling-pathway activation. In addition, mechanisms underlying the antidepressant-like effects of EA included both acupoint specificity and electrical stimulus. EA may serve as a potential tool for depression treatment in the future.

## Abbreviations

NSC: Adult neural stem cell; CUS: Chronic unpredictable stress; EA: Electroacupunture; NGF: Nerve growth factor; BDNF: Brain-derived neurotrophic factor; GDNF: Glial cell line-derived neurotrophic factor; ERK: Extracellular signal-regulated kinase; DG: Dentate gyrus; FST: Forced swimming test; EPM: Elevated plus-maze; PFA: Paraformaldehyde.

## Competing interests

The authors hereby declared that there is no competing interest.

## Authors’ contributions

JY and GW guided this study. JY, LY and QL designed the study. LY wrote the protocol, managed the literature searches, undertook the experiment operation and statistical analysis. LY wrote the first draft of the manuscript; QL and JY helped to revise the manuscript. NY, XZ and QH (these authors contributed equally to this work) contributed to some work of the statistical analysis and have approved the final manuscript.

## Pre-publication history

The pre-publication history for this paper can be accessed here:

http://www.biomedcentral.com/1472-6882/13/288/prepub
